# Incidence of mortality and its predictors among HIV-infected children receiving antiretroviral therapy in Amhara region: a multicenter retrospective follow-up study

**DOI:** 10.1186/s13052-025-01872-5

**Published:** 2025-03-07

**Authors:** Gebrehiwot Berie Mekonnen, Sileshi Mulatu, Bruck Tesfaye Legesse, Mengistu Abebe Messelu, Fikadie Dagnew Baye, Birara Ayichew Tilaye, Mengistu Melak Fekadie, Tiruye Azene Demile, Asnake Gashaw Belayneh, Sosina Tamre Mamo, Yeshimebet Tamir Tsehay, Ousman Adal, Betelhem Amha Haile, Birhanu Mengist Munie, Abraham Tsedalu Amare, Bekalu Mekonen Belay, Wubet Tazeb Wondie

**Affiliations:** 1 Department of Pediatrics and Child Health Nursing, College of Health Sciences, Debre Tabor University, Debre Tabor, Ethiopia; 2 Department of Pediatrics and Child health nursing, School of Health Science, College of Medicine and Health Science, Bahir Dar University, Bahir Dar, Ethiopia; 3 Department of Pediatrics and Neonatal Nursing, School of Nursing and Midwifery, Institutes of Health Science, Wollega University, Nekemte, Ethiopia; 4 Department of Nursing, College of Medicine and Health Sciences, Debre Markos University, Debre Markos, Ethiopia; 5 Department of surgical Nursing, School of Nursing, College of Medicine and Health Sciences, University of Gondar, Gondar, Ethiopia; 6 Department of Emergency and Critical Care Nursing, College of Medicine Health Science, Bahir Dar University, Bahir Dar, Ethiopia; 7 Department of Surgical Nursing, School of Health Science, College of Medicine and Health Science, Bahir Dar University, Bahir Dar, Ethiopia; 8 Department of Emergency, College of Medicine and Health Sciences, Bahir Dar University, Bahir Dar, Ethiopia; 9 Department of Human Nutrition, Institute of Public Health, College of Medicine and Health Sciences, University of Gondar, Gondar, Ethiopia; 10 Department of Psychiatry, College of Health Sciences, Debre Tabor University, Debre Tabor, Ethiopia; 11 Department of Adult Health Nursing, College of Health Sciences, Debre Tabor University, Debre Tabor, Ethiopia; 12 Department of Pediatrics and Child Health Nursing, College of Medicine and Health Science, Ambo University, Ambo, Ethiopia

**Keywords:** Incidence, Ethiopia, HIV infected children, Mortality, Predictors, WHO staging

## Abstract

**Background:**

Evidence shows that earlier access to Antiretroviral Therapy (ART) helps to increase the survival of children by delaying the progression to advanced stages of HIV-related diseases. However, the effect of testing and treatment strategies on mortality among children receiving ART has remained a limited study in Ethiopia. This study aimed to assess the incidence of mortality and its predictors among HIV-infected children receiving ART in Amhara Region Specialized Hospitals, after the test and treat strategy.

**Methods:**

A multicenter facility-based retrospective follow-up study was conducted on 475 HIV-infected children receiving ART at Amhara Region Comprehensive Specialized Hospitals from June 10, 2014, to February 28, 2022. A simple random sampling technique was used to select the study participants. Data were collected using national antiretroviral intake and follow-up forms via the KoBo Toolbox. Data analysis was done using STATA version 17. Descriptive analyses were summarized using the Kaplan-Meier curve, and a log-rank test was used to estimate and compare. Both bivariable and multivariable Weibull regression model were fitted to identify predictors of mortality. Finally, an adjusted hazard ratio with 95% CI was computed, and variables having a p-value < 0.05 were considered as statistically significant predictors of mortality.

**Results:**

Among the 461 (97.1%) records included in the final analysis [42], 9.11% of the individuals died within the follow-up period. In this study, the overall mortality rate was found to be 2.53 per 100 child-year observations (95% Confidence Interval (CI): 1.87, 3.43). HIV-infected children presenting with opportunistic infections (OIs) other than tuberculosis infection (adjusted hazard ratio (AHR): 3.81, 95% CI: 1.66, 8.72), tuberculosis (AHR: 7.14, 95% CI: 2.86, 17.79), wasting (AHR: 2.83, 95% CI: 1.44, 5.56), and advanced disease staging (AHR: 4.02, 95% CI: 1.84, 8.78) were at higher risk of mortality.

**Conclusion:**

In this study, the mortality rate was high after the test-and-treat strategy. HIV-infected children presenting with OIs, advanced disease staging, and wasting were at higher risk of mortality. Therefore, to increase the survival rate for HIV-positive children, clinicians should place a strong emphasis on early screening, controlling OIs, and optimizing nutritional supplements.

**Supplementary Information:**

The online version contains supplementary material available at 10.1186/s13052-025-01872-5.

## Introduction

Human immunodeficiency virus (HIV) and acquired immunodeficiency syndrome (AIDS) have become global emergencies, one of the most serious infectious diseases, and a major problem [1]. HIV gradually weakens the immune system, and AIDS without treatment is one of the most common causes of morbidity and mortality worldwide [2].

Globally, 39 million people were living with HIV in 2022, of whom 1.8 million were children, and 1.3 million people became newly infected, and from these 630,000 people died from AIDS-related illnesses worldwide [3]. Globally, it is estimated that 2.5% of all child deaths are associated with HIV infection. Africa remains the region most heavily affected, with 5% of all child deaths associated with HIV infection [4]. Ethiopia is one of the HIV hard-hit countries with a prevalence of 1.1% [5]. It progresses very rapidly among infants and children. In the absence of ART, 33% of infants will die before their first birthday, and 50% will die before the age of two years [6].

The mortality rate among HIV-infected children varies globally. In Asia, the rate is 1.9 deaths per 100 child-years (CYO) [7], while in Congo, it is 3.4 CYO [8], 1.6 CYO in Zambia [9], 2.9 CYO in Zimbabwe [10], and 8.4 CYO in Kenya [11]. In Ethiopia, the rates of mortality for children on ART show considerable variation, ranging from 1.12 to 6.3 deaths per 100 CYO [12–19].

The burden of mortality among children with HIV/AIDS who are receiving antiretroviral treatment is influenced by a variety of factors. Nevertheless, a number of significant risk factors for death include low hemoglobin levels (< 10 g/dl) [13, 20–27], a low cluster of differentiation 4 (CD4) count or % below the threshold level [13, 22, 24–26], World Health Organization (WHO) clinical stage III and IV [22, 25–27], poor adherence to ART [22, 26], not taking cotrimoxazole preventive therapy (CPT) [24, 26], and severe wasting [22, 24, 27].

In the meantime, Ethiopia has adopted a test-and-treat strategy since June 10, 2014, for all children initiation of ART regardless of CD4 cell count and WHO clinical staging aiming at reduction of HIV-related mortality and morbidity [28]. Recently, the United Nations Programme on HIV/AIDS (UNAIDS) reports showed that the WHO and Ethiopia Federal Ministry of Health (FMOH) set a strategy to improve HIV care and treatment to achieve the goal of 95–95–95. The recent data showed in Ethiopia that this goal was known; their status, accessing treatment, and viral suppression were 63%, 91%, and 81%, respectively [3].

Ethiopia’s FMOH and WHO have coordinated approaches to reduce HIV-related mortality in children. By prioritizing early ART initiation, universal access to ART, prevention of mother-to- child transmission (PMTCT) programs, and integrated care for pediatric HIV, adherence support, community-based HIV initiatives, comprehensive care models, and strengthening health systems, both organizations seek to decrease pediatric HIV mortality and enhance the health and quality of life for children living with HIV [29, 30].

ART is a critical strategy in managing HIV, preventing death, and improving quality of life. It reduces mother-to-child transmission of HIV, lowers the financial burden associated with treating opportunistic infections and providing palliative care, and helps prevent the progression to AIDS. Additionally, ART enhances immune function and contributes to the prevention of new HIV infections [30, 31]. This study is supported by achieving the Ethiopian Health Sector Transformation Plan II (HSTP-II) goal of reducing HIV/AIDS and its complications by the end of 2025, as well as the targets of the Sustainable Development Goals (SDG) [32].

Despite the implementation of the test-and-treat approach, there is no clear evidence of the overall incidence of mortality in study areas. In addition, previous studies had not considered rapid initiation of ART specifically; dolutegravir (DTG) contained ART drugs, disclosure of the child, and HIV status of the parents as independent variables. Therefore, in this study these variables were incorporated as independent variables to determine the incidence of mortality.

This study will help health professionals or clinicians to increase the existing knowledge, prioritize identified predictor variables, and identify further interventions for mortality. The study will provide input to the program planners and decision-makers on HIV/AIDS care and support at various levels and also an input for the further baseline for interventional research for regional and national programmers. Therefore, this study aimed to assess the incidence of mortality and its predictors among HIV-infected children receiving antiretroviral therapy in Amhara Region Comprehensive Specialized Hospitals, 2022.

## Methods and materials

### Study design, area, and period

An institution-based retrospective follow-up study design was conducted from May 17 to June 15, 2022. The region, which covers 159,173.66 square kilometers, has 858 Health Centers, 3560 Health Posts, and 81 hospitals, and eight comprehensive specialized hospitals (CSHs). Study was conducted on these CSHs are University of Gondar, Felege Hiwot, Debre Markos, Debre Tabor, Dessie, Woldia, and Debre Birhan except Tibebe-Ghion CSH. According to the January 2022 Ethiopian Demographic and Health report, the total population projection of the region is estimated at 30,848,988. These hospitals provide multidimensional care, including surgical, medical, pediatrics, and maternal health services. Since 2005, these hospitals have provided free ART services as part of the National AIDS Control Programme.

### Source population

All HIV-infected children aged < 15 years on ART in Amhara Regional State Comprehensive Specialized Hospitals.

### Study population

All HIV-infected children newly enrolled on ART between June 10, 2014, and February 28, 2022, in Amhara Regional State Comprehensive Specialized Hospitals.

### Eligibility criteria

#### Inclusion criteria

All newly enrolled HIV-infected children who have been on ART at Amhara Regional State Comprehensive Specialized Hospitals during the study period.

#### Exclusion criteria

Records with unknown date of ART initiation and outcome status were excluded from this study.

### Sample size determination, sampling procedures, and sampling technique

The sample size was calculated using common significant predictor variables (CD4, WHO clinical staging, ART adherence level, and anemia). STATA version 17, cox proportional hazard model used for sample size determination through the following assumptions: Z$\:\alpha\:/2$ = is the critical value of a standard normal distributed variable at 5% significance level = 1.96; Z$\:\beta\:/2$ = is the critical value of a standard normal distributed variable at 20% = 0.84; the probability of the event was (0.067 and 0.046) [33, 34] and the probability of withdrawal was 0.1. From the calculated sample sizes, the largest sample size (475) was selected as a sample size for this study. (Supplementary Table 1) The sample was allocated proportionally for the seven Comprehensive Specialized Hospitals, and records were selected using simple random techniques.

### Study variables

#### Dependent variables

The incidence of mortality among HIV-infected children receiving ART during the follow-up time was considered as an event of interest. Children who did not develop the events until the end of follow-up were considered censored. Incidence of mortality was calculated by dividing all deaths by the total number of months of follow-up time across all children during follow-up time.

#### Independent variables

##### Socio-demographic characteristics

age, sex, residence, current parent’s status, educational status of the caregiver, HIV disclosure status, and marital status of the caregiver.

##### Baseline clinical, nutritional, and laboratory characteristics

CD4 count, WHO clinical staging, hemoglobin (Hgb) level, anthropometric indices, Tuberculosis (TB) infection, OIs other than TB, functional& developmental status.

##### ART and medication-related characteristics

Baseline ART regimen, DTG contained ART drugs, presence of regimen change, level of ART adherence, TPT, CPT, ART side effect, Initiation of ART within seven days.

### Operational definition

#### Children

Individuals with ages less than 15 years old [35].

#### Event

Death of children after the initiation of ART.

#### The survival time

was calculated in months using the time between the date of highly active antiretroviral therapy (HAART) initiation and the time of death (event), lost to follow up, transfer out, and live at the end of the study.

#### Censored

recorded when HIV-infected children transferred out to other health institutions, lost to follow-up or alive during ART follow-up.

#### LTFU

was recorded when HIV-infected children missed their appointments from one month to three months.

#### Stunting

if the child has a Height for Age (HFA) or length for age (LFA) Z-score less than − 2 SD [30].

#### Wasting

, if the weight for height (WFH) Z-score is less than − 2 SD for less than five years, or if Body mass index (BMI) for age Z-score is less than − 2 SD for greater than five years [30].

#### The level of adherence to ART

Good adherence is reported with compliance equal to or greater than 95% or ≤ 3 missed doses per month as documented by the ART health personnel; fair reflected 85–94% compliance and between 4 and 8 missing doses per month) as documented by the ART health personnel, and poorly reflected less than 85% compliance or ≥ 9 missed dose per month) as documented by the ART health personnel [35].

#### Child developmental status

was classified as appropriate (able to attain milestones for age), delayed (failure to attain milestones for age); and regression (loss of what has been attained for age) as documented by the ART health personnel [30].

#### The functional status

was classified as working(capable of going out of home and doing routine activities including daily work), Ambulatory (capable of self-care and going to the toilet unsupported), and Bed-ridden (cannot go even to the toilet unsupported) as documented by the ART health personnel [30].

#### Rapid initiation of ART

ART initiation care and support on the same day of HIV confirmation or within seven days [36].

#### Anemia (low hemoglobin level)

was defined as having a hemoglobin level of less than 10 mg/dl [37].

#### CD4 counts or percentage (%) below the threshold

is considered if the child had CD4 cell counts < 1500/ mm3 or 25% for age < 12 months, CD4 cell counts < 750/ mm3 or < 20% for age 12–35 months, CD4 cell counts < 350/mm3 or < 15% for age 36–59 months, and CD4 cell counts < 200/mm3 or < 15% for age ≥ 60 months [30].

#### Baseline data

Any laboratory tests obtained at the time of ART initiation were considered baseline data. However, if laboratory tests were not done during ART initiation, any laboratory tests were done within a month of ART initiation [38].

### Data collection tool and procedures

The data were collected from the ART intake form, follow-up form, and children`s charts using the data extraction tool adopted from Ethiopian ART guidelines [30]. The variables consist of socio-demographic, clinical, & laboratory, ART, and medication-related variables. Data were collected by seven bachelor’s degree nurses who had smartphones and who were familiar with the ART follow-up and taking basic ART training.

### Data quality control

The data extraction tool was pretested at 5% of the sample size two weeks before the actual data collection period at UoG CSH. Moreover, one-day onsite training was given on how to review ART follow-up and medical records, data collection methods, and the objective of the study for data collectors and supervisors. Data were collected using the KoBo toolbox, which was prepared with relevant restrictions by trained nurses working in hospitals. The checklist was checked for consistency and completeness before submission. Besides the data collector, the supervisor and principal investigators carefully monitored the entire data collection process and daily submission report.

### Data processing and analysis

Data were collected using the KoBo toolbox and exported to STATA version 17 statistical software packages used for analysis. Also, WHO anthro and WHO anthroPlus software were used to generate anthropometric indices. Descriptive statistics such as mean with SD and percentage and frequency were used to characterize the data. The incidence of deaths per 100 child-year observations (CYO) was calculated. The variance inflation factor (VIF) was used to check the association between predictor variables.

Kaplan-Meier failure curve was used to estimate death-survival probabilities. The log-rank test was employed to compare statistical differences between independent variables. The proportional hazard assumption (PHA) was checked using both graphical and statistically tests using a global test, and it revealed that the PHA was satisfied. The log-likelihood and Akaike Information Criteria (AIC) were applied to select the best-fitted model, and a model with minimum AIC was considered the best-fitted model.

Based on this, the Weibull regression model with the value was the best-fitted model. In addition, the goodness-of-model fitness was also checked using the Cox-Snell residual test. Variables having a *p* < 0.25 in the bivariable analysis were fitted into the multivariable Weibull regression model. Hazard ratio with 95% CI was used to determine the strength of the association. Variables having a *p* < 0.05 in multivariable analysis were considered statistically significant.

## Results

### Socio-demographic, baseline clinical, nutritional, and laboratory, ART and medication-related characteristics of HIV-infected children on ART

A total of 461 HIV-infected children’s medical records with a completeness rate of 97.1% participated in the study. The mean (± SD) age of children was 7.5 (± 4.03) years. 29% of the children were in the age group of < 5 years, 58.13% of the children were males. On parent characteristics, 54.88% were married, 33.19% of them had no formal education, and 70.5% of them were alive. The majority (79.39%) of children had CD4 cell count above the threshold, and about 16.05% had anemia; 30.8% and 48.9% nutritional statuses were wasted and stunted, respectively. Additionally, 7.16% of the study co-infected with tuberculosis. In this study, the magnitudes of good adherence to ART, intake of TPT and CPT during the follow-up period by the children were 70.5%, 63.77%, and 82.21%, respectively. In addition, only 52.93% of them initiated ART within seven days after admission. (Table 1) Based on functional status, 65.03% were working, and appropriate motor developmental status was 67.41% (Supplementary Table 2).

**Table 1:** Socio-demographic, baseline clinical, nutritional, and laboratory, ART and medication-related characteristics children on ART at Amhara Region Comprehensive Specialized hospitals, Ethiopia, 2022 (*n* = 461)

Characteristics	Frequency(*n*)	Percentage (%)
**Age (in years)**		
< 5	135	29.28
5–9	159	34.43
10–14	167	36.23
**Sex**		
Female	193	41.87
Male	268	58.13
**Residence**		
Rural	135	29.28
Urban	326	70.72
**Educational Status of the caregiver**		
No formal education	153	33.19
Primary	168	36.44
Secondary	73	15.84
Tertiary (College & above)	67	14.53
**Marital status of the caregiver**		
Married	253	54.88
Unmarried	95	20.61
Divorced	76	16.49
Widowed	37	8.03
**CD4 cell count (** *n* ** = 461)**		
Above threshold	366	79.29
Below threshold	95	20.61
**Anemia (** *n* ** = 461)**		
No	387	83.95
Yes	74	16.05
**Wasting (** *n* ** = 461)**		
No	319	69.2
Yes	142	30.8
**Stunting (** *n* ** = 461)**		
No	237	51.41
Yes	224	48.59
Presence of Opportunistic infections other than tuberculosis (*n* = 461)		
No	371	80.48
Yes	90	19.52
**Presence of Tuberculosis infections (** *n* ** = 461)**		
No	428	92.84
Yes	33	7.16
**WHO Clinical staging**		
Stage I	285	61.82
Stage II	92	19.96
Stage III	58	12.58
Stage IV	26	5.64
**ART drug adherence level**		
Good	325	70.5
Fair	74	16.05
Poor	62	13.45
**TPT taken**		
Yes	294	63.77
No	167	36.23
**CPT taken**		
Yes	379	82.21
No	82	17.79
**ART side effect**		
No	362	65.51
Yes	159	34.49
**Initiated ART within seven days**		
Yes	268	52.93
No	217	47.07

### Incidence of mortality among HIV-infected children during the follow-up period

Four hundred and sixty-one HIV-infected children on ART had a follow-up time from 1 to 93 months. The observation of total time at risk was 19896.00 child per month observations or 1658.00 child-year observations (CYO). From the total enrolled HIV-infected children on ART, during the follow-up 9.11% (95% CI: 6.79–12.11%) developed the event of interest (mortality), and 90.89% were censored. The overall incidence of mortality was 2.53 (95% CI; 1.87, 3.43) per 100 CYO. The mean survival time was 84.6 (95% CI: 82.19, 87.02) months. **Kaplan-Meier failure function**.

The free-probability of mortality for the total follow-up time by the end of study was 0.8778 (95% CI; 0.8314, 0.912), whereas it was 0.9554 (95% CI; 0.9317, 0.971), 0.9216 (95% CI; 0.8914, 0.9437), and 0.8743 (95% CI; 0.8311, 0.912), at the end of 6 months, 2 years, 3 years, and 4 years, respectively.

### Comparison of death-free survival probability for different categorical variables

**Fig. 1:**
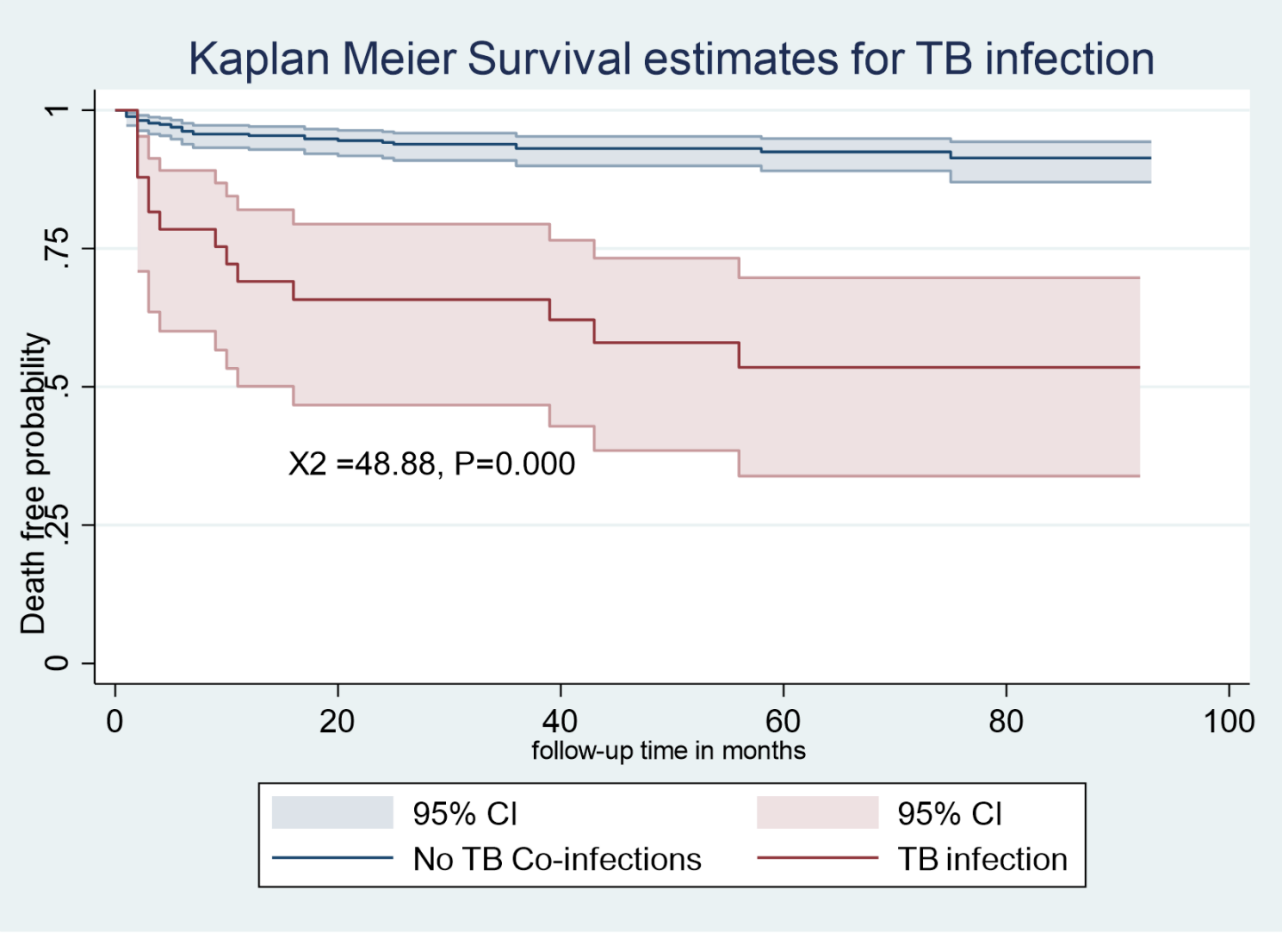
Kaplan-Meier death-free survival probability among HIV-infected children receiving ART based on TB infection in Amhara regional state comprehensive specialized hospitals, Ethiopia, 2022

**Fig. 2:**
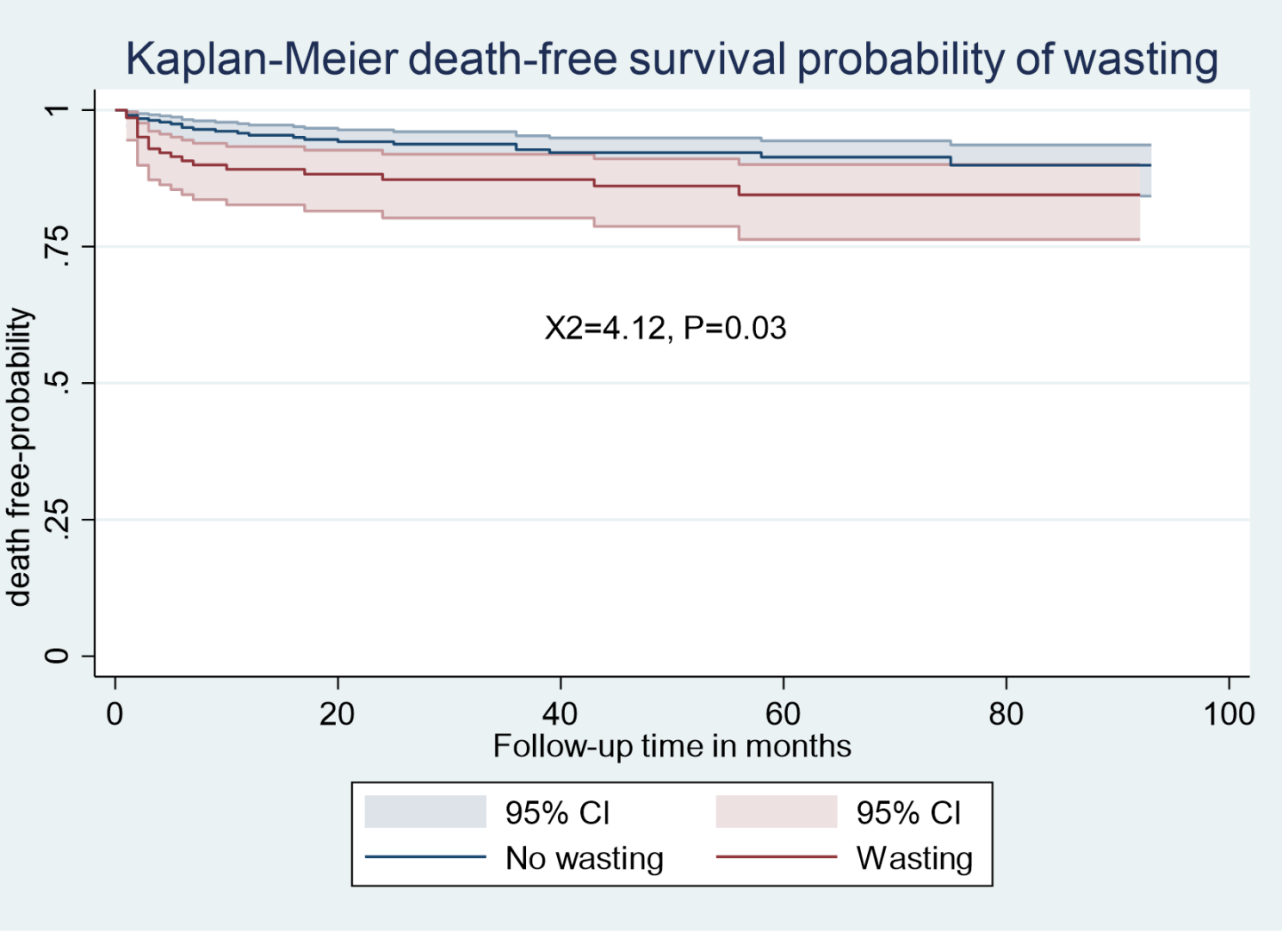
Kaplan-Meier death-free survival probability among HIV-infected children receiving ART based on nutritional status in Amhara regional state comprehensive specialized hospitals, Ethiopia, 2022

**Fig. 3:**
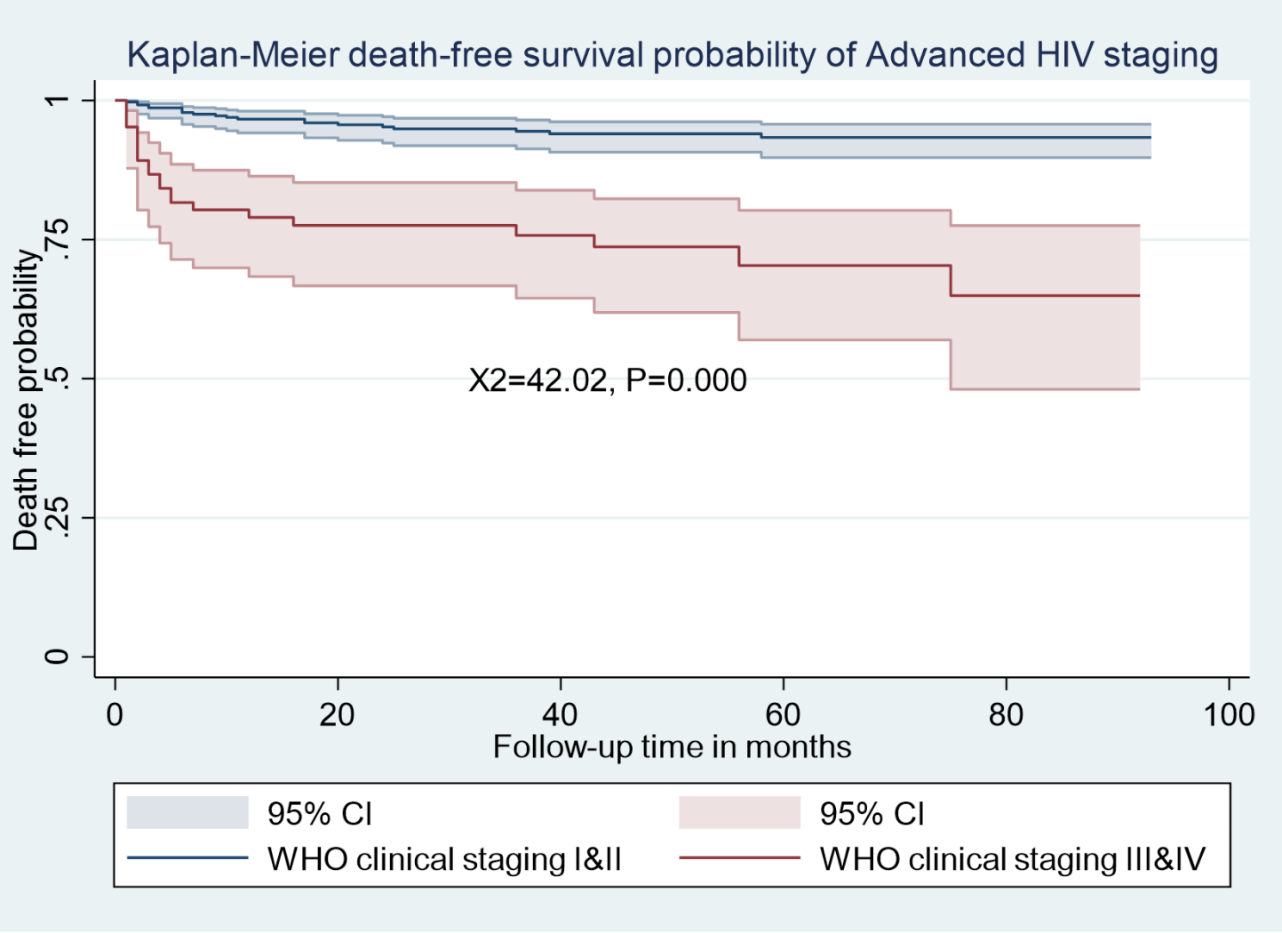
Kaplan-Meier death-free survival probability among HIV-infected children receiving ART based on advanced HIV staging in Amhara Regional State Comprehensive Specialized Hospitals, Ethiopia, 2022

**Fig. 4:**
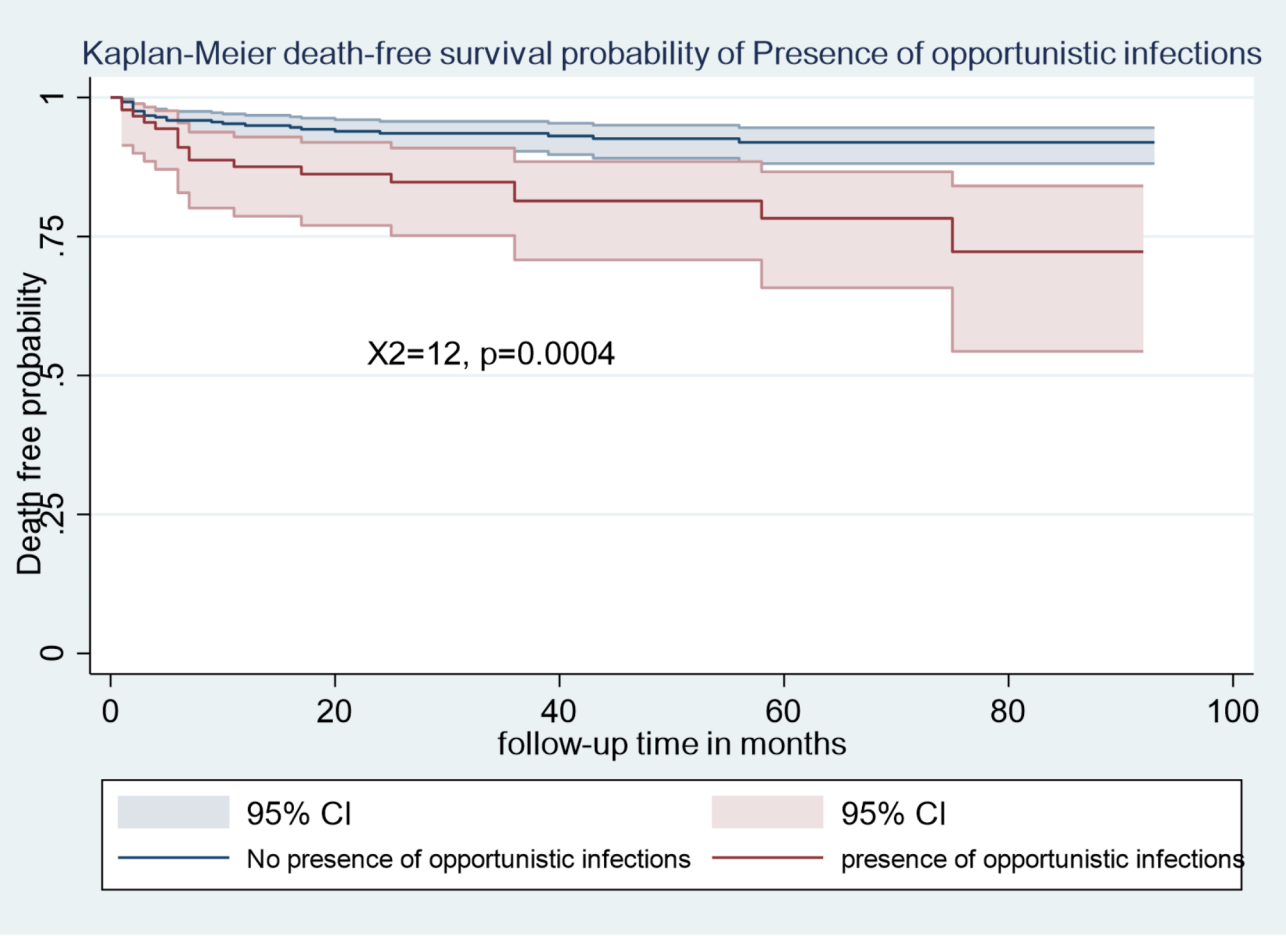
Kaplan-Meier death-free survival probability among HIV-infected children receiving ART based on presence of opportunistic infections in Amhara Regional state comprehensive specialized hospitals, Ethiopia, 2022

The Kaplan-Meier survival estimation graphs **(**Figs. 1, 2, 3 and 4**) display** death-free survival probabilities among children receiving ART, stratified by baseline WHO clinical stage, TB co-infection status, nutritional status, and presence of opportunistic infections other than TB. For example, having free from TB infection were lower death -free survival probability compared to children who had TB infection (log-rank X^2^ = 48.88, *p* = 0.0000).

### Proportional hazard assumption (PHA) test and model comparison

The PHA was checked using the Schoenfeld residuals test. The test showed that the p-value for each covariate and the whole covariates simultaneously were above 0.05 (X^2^ = 19.25, *p* = 0.2027). To select the best model for the data set, a comparison of the semi-parametric and parametric hazard models was done statistically using Akakie and Bayesian Information Criterion (AIC, BIC) and graphically using the Cox-Snell residual test.

Based on this, the Weibull regression model (AIC = 307.22), was the best model than the other parametric and semi-parametric Cox proportional hazard models. So, all of the interpretations in this study were using the Weibull regression model.

### Predictors for incidence of mortality among HIV-infected children on ART in Amhara regional state

In the bivariable analysis, variables under study were predictors such as age, marital status, parental status of the care givers, educational status of the care givers, baseline WHO clinical staging, baseline CD4 count, baseline hemoglobin status, functional & developmental status, regimen change, ever taking TPT, ever taking CPT, ART adherence level, TB co-infection, and presence of OI other than TB infection. However, in the multivariable Weibull regression analysis, only factors such as WHO clinical staging, TB co-infection, and the presence of OIs other than TB infection were significant predictors for the incidence of mortality among HIV-infected children on ART.

We observed that the hazard of death among children presented with WHO clinical staging III&IV was 4.02 times [AHR: 4.02 (95% CI: 1.84, 8.78)] higher than as compared to those children with WHO clinical staging I & II. Additionally, the hazard of death among children who initiated ART with wasting was 2.83 times [AHR: 2.83 (95% CI: 1.44, 5.56)], more likely as compared to their well-nourished counterparts.

Furthermore, the hazard of death among children with TB co-infection is 7.14 times [AHR: 7.14 (95% CI: 2.86, 17.79)] higher as compared to non-TB children. Lastly, the hazard of death among HIV-infected children who had the presence of OIs other than TB infections was 3.81 times [AHR: 3.81 (95% CI: 1.44, 5.56)] as compared to their counterparts. **(**Table 2**)**

**Table 2:** A bivariable and multivariable Weibull regression analysis of predictors of mortality among children on ART in Amhara region comprehensive specialized hospital, 2022 (*n* = 461)

Variables	**Status**		CMO	Death IDR per 100 CYO (95% CI)	CHR (95% CI)	**AHR (95% CI)**
	Event	Censored				
**Age**						
< 5 years	19	116	4748	4.8 (3.06, 7.53)	2.21(1.09, 4.47)	1.46(0.53, 3.98)
5 to 10 years	10	149	7252	1.65(0.85, 3.08)	0.82 (0.36, 1.87)	0.78(0.32, 1.89)
> 10 years	13	154	7896	1.98 (1.15, 3.4)	**1**	**1**
**Marital status of the caregiver**						
Married	16	237	11449	1.68(1.03, 2.74)	**1**	**1**
Unmarried	9	86	3729	2.9 (1.51, 5.57)	1.64 (0.73, 3.72)	2.18 (0.88, 5.38)
Divorced	11	65	3045	4.33 (2.4, 7.83)	2.49 (1.16, 5.37)	2.17 (0.85, 5.52)
Widowed	6	31	1675	4.3 (1.93, 9.58)	2.57 (1.01, 6.57)	1.62 (0.55, 4.72)
**Parental status of the child**						
Both parents alive	24	301	14477	1.99(1.33, 2.97)	**1**	**1**
One or both parents dead	18	118	5419	3.99(2.51, 6.33)	1.93 (1.04, 3.55)	1.05 (0.47, 2.34)
**Educational status of the caregiver**						
No formal education	18	135	5973	3.62(2.28, 5.74)	2.96 (0.87, 10.07)	1.28 (0.35, 4.64)
Primary	15	153	7793	2.31(1.39, 3.83)	2.01 (0.58, 6.93)	0.63 (0.16, 2.45)
Secondary	6	67	3023	2.38 (1.07, 5.3)	1.97 (0.49, 7.9)	1.11 (0.25, 4.89)
Tertiary(College& above)	3	64	3107	1.16(0.37, 3.59)	**1**	**1**
**WHO Clinical staging**						
Stage I & II	20	357	16873	1.42 (0.92, 2.2)	**1**	**1**
Stage III & IV	**22**	**62**	**3023**	**8.73(5.75, 13.26)**	**5.82 (3.17, 10.66)**	4.02(1.84,8.78)***
**CD4 cell count**						
Above threshold	25	341	16580	1.81(1.22, 2.68)	**1**	**1**
Below threshold	17	78	**3316**	**6.15(3.82, 9.90)**	3.15 (1.69, 5.83)	0.85 (0.34, 2.08)
**Anemia**						
No	22	365	17250	1.53(1.01, 2.32)	**1**	**1**
Yes	20	54	**2646**	**9.07(5.85,14.06)**	5.53(3.01,10.13)	1.81 (0.83, 3.94)
**Working and appropriate motor developmental status**						
Yes	18	287	13592	1.59 (1.00, 2.52)	**1**	**1**
No	24	132	6304	4.57 (3.06, 6.82)	2.75 (1.49, 5.07)	1.79 (0.92, 3.49)
**Adherence level of ART**						
Good	23	302	14439	1.91 (1.27, 2.88)	**1**	**1**
Fair& poor	19	117	5457	4.18 (2.67, 6.55)	2.09 (1.14, 3.84)	0.84 (0.37, 1.9)
**ART regimen change**						
Yes	19	215	11618	1.91(1.25, 3.08)	**1**	**1**
No	23	204	8278	3.33 (2.22, 5.02)	1.53 (0.83, 2.82)	1.02 (0.51, 2.06)
**Ever taking tuberculosis preventive therapy**						
Yes	19	275	14439	1.91 (1.27, 2.88)	**1**	**1**
No	23	144	5457	4.18(2.67,6.55)	2.27 (1.24, 4.18)	0.74 (0.34, 1.59)
**Ever taking cotrimoxazole preventive therapy**						
Yes	30	349	16721	2.15 (1.51, 3.08)	**1**	**1**
No	12	70	3175	4.54 (2.58, 7.99)	2.02 (1.03, 3.95)	1.05 (0.46, 2.37)
**Wasting**						
No	23	296	13856	1.99 (1.32, 3.00)	**1**	**1**
Yes	19	123	6040	3.77 (2.41, 5.92)	**1.91 (1.04, 3.5)**	2.83 (1.44, 5.56)**
**Presence of opportunistic infections other than Tuberculosis**						
No	25	346	16237	1.85 (1.25, 2.73)	**1**	**1**
Yes	17	73	**3659**	**5.58 (3.47, 8.97)**	**2.91 (1.56, 5.38)**	3.81 (1.44, 5.56)**
**Tuberculosis co-infection**						
No	28	400	18574	1.81 (1.25, 2.62)	**1**	**1**
Yes	**14**	**19**	**1322**	12.71(7.53,21.4)	**7.08(3.77,13.44)**	7.14(2.86, 17.79)***

Incidence density rates (IDRs) per 100 child-years of observation were calculated for each category. Log-rank tests were used to compare mortality rates between groups. IDR = Incidence density rate, CRO = Child-year observation, CMO = Child month observation, * Significant at α 0.05, 1: reference, CHR, crude hazard ratio, AHR, Adjusted hazard ratio

## Discussion

This study identified the incidence and predictors of mortality among HIV-positive children receiving ART using a multicenter facility-based retrospective cohort study in Amhara region comprehensive specialized hospitals.

At the end of follow-up, about 9.11% of patients were deceased. The overall incidence of mortality rate of this study was 2.53 per 100 CYO (95% CI: 1.87, 3.43), which is aligning with findings from various regions: 1.9 deaths per 100 CYO in Asian Pacific region [7], 3.0 deaths per 100 CYO in Andhara Pradesh, India [39], 3.4 per 100 CYO in the Congo [8], 2.9 per 100 CYO in Zimbabwe [10], and between 2.56 and 4.4 deaths per 100 CYO in Ethiopia [24, 26].

Conversely, the mortality rate found in this study is much lower than a Kenyan study where the findings reflected 8.4 deaths per 100 CYO [11], 4 deaths per 100 CYO in Bahir Dar [14], and in Debre Tabor and Dessie specialized hospital 6.3 deaths per 100 CYO [22]. The explanations for variation in the incidence of mortality rate might be due to the difference in sample size, study settings, study period, and participant characteristics. The higher mortality rate in this study may relate to the clinical profile of participants; specifically, 20% had advanced HIV/AIDS with severe immunodeficiency, as indicated by low CD4 counts, at the time of ART initiation. Additionally, as the study was conducted in referral hospitals, participants often presented with advanced disease and complex management needs, factors that likely contribute to increased mortality [40].

However, this finding is higher than studies conducted in 1.40 per 1000 CYO in Mekele, Northern Ethiopia [18], and 0.97 per 100 CYO in Nigeria [41]. It might be due to the associated effect of INH which prevents the occurrence of OIs like TB and life-threatening bacterial infections. In our study, 36.23% and 17.79% of TPT and CPT were not taken as prophylaxis, and the difference could be a longer follow-up period than the study conducted previously. CPT and TPT prevent the development of very serious and fatal OIs, and hence they contribute to lower mortality and morbidity [42].

This finding is higher than those reported in studies from Mekele, Northern Ethiopia [18], Northern Ethiopia (1.40 per 1000 person-months of observation), and Nigeria (0.97 per 1000 person-months of observation) [40]. The difference may be due to the protective effect of isoniazid (INH) in preventing OIs such as TB and severe bacterial infections. In our study, 36.23% and 17.79% of participants did not receive TPT and CPT prophylaxis, respectively. Additionally, the longer follow-up period may account for the higher rates observed, as CPT and TPT are known to prevent severe and fatal OIs, thereby reducing mortality and morbidity [41].

In this study, the hazard of mortality is 2.57 times high on HIV-infected children who had been severely malnourished during the commencing ART. This finding is consistent with studies conducted in the Asia Pacific region [43], Zambia [9], Tanzania [27], Ghana [44], and Ethiopia [19, 22, 23]. Generally, severe acute malnutrition in HIV patients is complex, with nutritional deficiencies adding risk, especially in immune-suppressed children. In the context of this study, in which more than one fifth of children classified in the immune suppressed category, wasting would contribute to an additional risk of death due to nutritional deficiency. HIV can cause malnutrition through diarrhea, while malnutrition weakens infection resistance, potentially accelerating HIV progression. Children with advanced HIV/AIDS and severe malnutrition face limited immune recovery and heightened vulnerability to severe opportunistic infections [45].

Additionally, wasting is high risk of death in HIV-infected children due to reduced caloric intake, gastrointestinal dysfunction, or metabolic abnormalities independent of abnormal energy expenditure, and also therapy related reasons to have gastrointestinal symptoms, which may predispose them to wasting and result in poor recovery [46, 47]. Good nutrition has been proven to increase resistance to infection and disease and improve energy, and the reverse is true [48]. HIV decreases food consumption as a result of swallowing difficulty caused by oral trash or esophageal candidiasis, reduces nutritional absorption caused by gastrointestinal mucosal damage, and increases the metabolic demands [23]. Consequently, this will increase the risk of death of children on ART [49, 50].

Children with advanced WHO HIV clinical stage (III and IV) at the time of ART initiation have 2.87 times a higher risk of death as compared to their counterparts with mild status (i.e., WHO HIV clinical disease stage (I and II)). This finding is highly supported by previous studies conducted in Ethiopia [13, 15–19], Zambia [9], Kenya [51], Malawi [52], Cameroon [53] and Zimbabwe [10] which all indicated that advanced WHO HIV clinical disease stages were a predictor of mortality. For HIV-positive children, the risk of developing and recurring OIs rises with advancing WHO HIV clinical stages. OIs are the most common morbidity among children in advanced WHO stages, indicating that as clinical staging progresses, the likelihood of OIs increases, potentially contributing to mortality.

Furthermore, the hazard of death among children with TB co-infection is 7.14 times higher as compared to non-TB children. And the hazard of death among HIV-infected children who had the presence of an opportunistic infection other than TB infections was 3.81 as compared to those of their counterparts. This finding was supported by the findings of a study conducted in South Africa [54], Tanzania [55], Nigeria [41], and Ethiopia [26, 56].

The present study does have some inherent limitations. Due to financial constraints, incidence and predictors of mortality after initiation of ART were assessed retrospectively instead of prospectively. As data were collected from secondary sources, incompleteness, loss, or transfer out was inevitable, and it was difficult to assess clinical and immunological responses. As a result, the study may fail to assess possible causes of death.

### Conclusions and recommendation

In this study, the mortality rate was high. OIs advanced disease staging (III and IV), and wasting were factors found to be significantly increasing the risk of mortality among HIV-infected children receiving ART. Therefore, clinicians should emphasize early screening, managing OIs, and maximizing nutritional supplements for HIV infected children to improve the survival rate of those individuals. Additionally, clinicians should give more emphasis to malnourishment in HIV-infected children at ART initiation. Furthermore, greater attention and close follow-up shall be given for HIV-infected children in the early ART phase. Comprehensive specialized hospitals shall closely monitor children to come up with baseline clinical variables. Further prospective cohort study shall be conducted by incorporating important predictors of mortality like income status, caregiver of the occupational status, family size, and viral load.

## Electronic supplementary material

Below is the link to the electronic supplementary material.

Supplementary Material 1

Supplementary Material 2

Supplementary Material 3

## Data Availability

The datasets generated during the current study are not publicly available due to confidentiality issues since the study was conducted among HIV-infected children. But data will be available upon reasonable request from the corresponding author.

## Abbreviations

AHR: Adjusted Hazard Ratio
AIDS: Acquired Immune Deficiency Syndrome
ART: Antiretroviral Therapy
CD4: Cluster of Differentiation 4
CPT: Cotrimoxazole Preventive Therapy
CI: Confidence interval
CSHs: Comprehensive Specialized Hospitals
CYO: Child-Year-Observations
HIV: Human Immunodeficiency Virus
HSTP-II: Health Sector Transformation plan-II
OIs: Opportunistic Infections
SD: Standard Deviation
TB: Tuberculosis
TPT: Tuberculosis Preventive Therapy
UoG: University of Gondar
WHO: World Health Organization
AIC: Akakie Information Criterion
BIC: Bayesian Information Criterion
PHA: proportional hazard assumption
UNAIDS: The Joint United Nations Programmme on HIV/AIDS
DTG: Dolutegravir
