# Supplementary material for: Incidence of mortality and its predictors among HIV-infected children receiving antiretroviral therapy in Amhara region: a multicenter retrospective follow-up study
Source: Ital J Pediatr. 2025 Mar 7;51:68. doi: 10.1186/s13052-025-01872-5 (PMC11887065; doi:10.1186/s13052-025-01872-5)
Supplement: Supplementary file 2 — Supplementary Material 2 [file 13052_2025_1872_MOESM2_ESM.docx]

Supplementary Table 1: Estimated sample size determination by predictor variables for incidence of mortality among HIV-infected children on ART, by using STATA version 17, cox-model

| Variables | AHR | Power | Probability of withdrawal | Probability of event | Sample size (n) |
| --- | --- | --- | --- | --- | --- |
| **CD4** | 3.4 | 80% | 0.1 | 0.067 | 348 |
| **WHO clinical staging** | 4.8 | 80% | 0.1 | 0.067 | 212 |
| **ART adherence** | 3.91 | 80% | 0.1 | 0.046 | 408 |
| **Anemia** | 3.54 | 80% | 0.1 | 0.046 | 475 |
