# Supplementary material for: Incidence of mortality and its predictors among HIV-infected children receiving antiretroviral therapy in Amhara region: a multicenter retrospective follow-up study
Source: Ital J Pediatr. 2025 Mar 7;51:68. doi: 10.1186/s13052-025-01872-5 (PMC11887065; doi:10.1186/s13052-025-01872-5)
Supplement: Supplementary file 3 — Supplementary Material 3 [file 13052_2025_1872_MOESM3_ESM.docx]

Supplementary table 2: Baseline clinical, ART and medication-related characteristics children on ART at Amhara Region Comprehensive Specialized Hospitals, Ethiopia, 2022 (n=461)

| **Characteristics** | **Frequency(n)** | **Percentage (%)** |
| --- | --- | --- |
| **Current parent status** | | |
| Both parents alive | 325 | 70.5 |
| One parent alive | 118 | 25.6 |
| Both parents deceased | 18 | 3.9 |
| **HIV status of the parents** | | |
| Non-reactive | 173 | 37.53 |
| Reactive | 288 | 62.47 |
| **Disclosure status the child** | | |
| Yes | 242 | 52.49 |
| No | 219 | 47.51 |
| **Functional status (n=326)** | | |
| Working | 213 | 65.34 |
| Ambulatory | 110 | 33.74 |
| Bedridden | 3 | 0.92 |
| **Developmental status (n=135)** | | |
| Appropriate | 92 | 68.15 |
| Delayed | 38 | 27.41 |
| Regressed | 6 | 4.44 |
| **Presence regimen change** | | |
| Yes | 234 | 50.76 |
| No | 227 | 49.24 |
| **Baseline ART drug regimen** | | |
| AZT-3TC-NVP | 99 | 21.48 |
| AZT+3TC+EFV | 93 | 20.82 |
| AZT+3TC+LPV/r | 22 | 4.77 |
| ABC+3TC+LPV/r | 65 | 14.1 |
| ABC+3TC+DTG | 62 | 13.45 |
| ABC+3TC+EFV | 57 | 12.36 |
| ABC+3TC+NVP | 25 | 5.42 |
| TDF+3TC+EFV | 22 | 4.77 |
| TDF+3TC+DTG | 13 | 2.82 |
| **DTG contained ART drugs** | | |
| Yes | 75 | 16.27 |
| No | 386 | 83.73 |
