# Supplementary material for: Incidence of mortality and its predictors among HIV-infected children receiving antiretroviral therapy in Amhara region: a multicenter retrospective follow-up study
Source: Ital J Pediatr. 2025 Mar 7;51:68. doi: 10.1186/s13052-025-01872-5 (PMC11887065; doi:10.1186/s13052-025-01872-5)
Supplement: Supplementary file 1 — Supplementary Material 1 [file 13052_2025_1872_MOESM1_ESM.docx]

**Dear, Professor Carlo Caffarelli**
Editorial Board Member, Italian Journal of Pediatrics

We would like to express our sincere gratitude to the editor and reviewers for reviewing our manuscript and providing valuable feedback. Your insightful remarks and recommendations have been beneficial. We have addressed the comment in detail (point by point)a.

**Reviewer Comments:**

**Reviewer #2:** On page 4, lines 119 to 125 are similar to the next paragraph (Lines 126 to 133). The second paragraph needs to be deleted.

**Author's response: Thank you in advance for your valuable comments. Based on your recommendation comments are accepted and revised in the main manuscript.**
